# Red Water

**Published:** 1901-09

**Authors:** J. M. Gibb

**Affiliations:** Government Inspector of Stock, Kingston, Jamaica


### REPORTS OF CASES.

### Red Water.

By J. M. Gibb, V.S.,
GOVERNMENT INSPECTOR OF STOCK, KINGSTON, JAMAICA.

As I have been consulted on four occasions beside treating two cases of my own of "red water" in imported cows, and as you might, together with the assistance of the numerous readers of your Journal, be able to throw some light upon the true cause and pathology of what appears to be out here a most fatal malady, I will now give you the history of the consulted cases and that with treatment of one of my own.

In the first case I was called to the country, and found my patients with temperatures between 105° F. and 107° F. They ate and chewed their cud, the former but slightly. Their bowels appeared fairly normal, and the urine also. The usual saline purge was administered, accompanied with antipyretics and febrifuges. The disease advanced, notwithstanding, until the period of red water arrived, when the strength of the animal became lowered and lowered until death closed the scene.

I then made up my mind to treat the next case in a different manner. Unfortunately, this was my own! One morning my boy reported that the cow had not eaten all her grass with which she had been fed overnight. I looked at her and noticed a dull appearance of the eyes and hanging ears. I took her temperature, and found it 107° F. I immediately gave salts, followed with salicylates and sulphites, keeping her strength up with stimulants (whiskey). This was on a Tuesday morning, and on Saturday morning following the temperature rapidly fell, the cow dying on Sunday afternoon. I made no post-mortem. Another case of my own, which I will give in its entirety, will show to you the persistency of the temperature.

On the morning of April 6th my boy reported that a cow had not eaten all her grass and would not touch her feed. I took her temperature, and found it 107° F. Instead of her usual yield of five quarts of milk this was reduced to two quarts. She looked dull, and her ears hung. There was slight yellowness of conjunctival membranes; bowels loose; urine of ordinary color. I gave her one-half pound of Epsom salts, ginger, sodii sulphis, ℥iv.; sodæ bicarb., ℥iv.; pot. nit., acid. carbolic., ♏xxx. Gruel.

5 P.M., temperature $105\frac{2}{5}$°. ℞.—Liq. amm. acet. C.°, ʒiv.; sprts. æth. nit., ℥ij.; acid. carbolic., ♏xxx. Gruel.

7th, 6 A.M., temperature 105°; 8.30 A.M., temperature 107°. ℞.—Epsom salts, ℥iv.; sodii sulphis, ʒiv.; sodæ salicylas, ʒiss.; acid. carbolic., ♏xl. Gruel.

Urine clear; dung not very healthy in consistence; ate bran mash; no desire for green grass; picks hay; eyes distressed; ears hanging; skin hot; breathing accelerated.

12 noon. Temperature, 107° F. Bowels acted twice since morning. ℞.—Hydrarg. subchlor., ʒj. on tongue. Liq. amm. acet. C.°, ʒiv.; sprts. æth. nit., ℥ij.; li. aconite (Flemming's), ♏xl. Gruel.

Munched a little hay and grass and drank a couple of quarts of water.

4.30 P.M., temperature 105°. ℞.—acid. carbolic., ♏xl.; sodii sulphis, ʒiv.; pot. bicarb., ʒiij. Gruel.

8.30 P.M., temperature 105°. ℞.—Liq. amm. acet. C.°, ʒiv.; sprts. æth. nit., ℥ij.; acid. carbolic., ♏xxx. Gruel.

8th, 6 A.M., temperature $105\frac{2}{5}$°. Ate very little; has not drunk any water; bowels acted sparingly. ℞.—Sodii sulphis, ʒij.; pot. bicarb., ʒiv.; acid. carbolic., ♏xxx. Gruel.

7.30 A.M., temperature $105\frac{4}{5}$°. ℞.—Ol. tereb., ℥j.; acetanilid, ʒiv.; proof rum, ℥ij.; sodæ salicylas, ʒij.; ol. lini., ℥xx.

10 A.M., temperature $102\frac{2}{5}$°.

12 noon. Temperature $101\frac{3}{5}$°. ℞.—℥j. proof rum in gruel; also acetanilid, ʒj. Sodæ salicylas, ʒj. Has been chewing cud.

6 P.M., temperature 102°. Ate four quarts of feed; still dull.

9 P.M., temperature 102°. ℞.—Pot. bicarb., ʒij.; pot. nit., ʒiv.; tr. cinchon., ℥j. Gruel.

9th, 6 A.M., temperature 105°.

7 A.M., temperature $107\frac{1}{5}$°. ℞.—Acetanilid, ʒiv.; sodæ salicyl., ʒij. Gruel. Refused feed.

9.15 A.M., temperature 106° F. ℞.—Proof rum, ℥ij.; acetanilid, ʒij.; sodæ salicyl., ʒiss. Gruel.

1 P.M., temperature $105\frac{3}{5}$°. Chews cud. ℞.—Liq. amm. acet. C.°, ʒiv.; sprts. æth. nit., ℥ij.; tr. aconite, ♏xx.; acid. carbolic., ♏xxx. Gruel.

4.30 P.M., temperature $104\frac{1}{5}$°. Gave rectal injection. ℞.—Liq. amm. acet. C.°, ʒiv.; sprts. æth. nit., ℥ij. Gruel.

9 P.M., temperature 105°. ℞.—Quin. sulph., acetanilid, of each ʒij. Gruel.

10th, 6 A.M., temperature 104°. Above repeated. Bowels evacuated during night; very yellow and unhealthy in look and odor.

10.30 A.M. Temperature, 106°. ℞.—Sodii sulphis, ʒij.; pot. bicarb., ʒiv.; acid. carbolic., ♏xxx.; proof rum, ℥ij. Gruel.

1 P.M., temperature $106\frac{3}{5}$°. Saline purgative, $\frac{1}{2}$ pound. Micturated dark urine for the first time; will not eat or drink. Gave proof rum in gruel.

3.30 P.M. Drank a bucket of water. Temperature, 106°.

5 P.M., temperature 105°. Gave proof rum in gruel.

7 P.M., temperature 105°. Gruel.

10 P.M., temperature 105°. Same with rum.

11th, 6 A.M., temperature 105°. Bowels acted once during night.

7 A.M., temperature $105\frac{3}{5}$°. Gruel. ℞.—Anisi. soda, pot. nit., ginger, quinine.

9 A.M., temperature $105\frac{4}{5}$°. Rum, acid. carb., gruel.

12 noon. Temperature $105\frac{2}{5}$°. Gruel.

1.30 P.M., temperature 106°. ℞.—Amm. chlor., ʒiv.; sprts. æth. nit., ℥j.; tr. aconite, ♏xl. Gruel.

3.30 P.M., gruel.

6 P.M., temperature $105\frac{1}{5}$°. Rum in gruel.

10 P.M. ℞.—Amm. chlor., ʒvi.; sprts. æth. nit., tr. aconite, ♏xl.; acid. carb., ♏x. Gruel.

12th, 6 A.M., temperature 106°. Gruel.

Some action of bowels; gave enema of warm water; although weak, looks lively; breathing fairly regular; pulse 96; drank a little water.

8 A.M. ℞.—Amm. chlor., ʒvi.; rum, ℥j.; acid. carbolic., ♏xx. Gruel.

3 P.M, temperature 107°. Gruel.

5 P.M. ℞.—Amm. chlor., ʒij.; rum, ℥ij.; sodæ salicyl., ʒiij. Gruel.

13th, 6 A M., temperature 106°. Gruel.

7.30 A.M. ℞.—Amm. chlor., ʒiv.; sodæ salicyl., ʒij.; rum, ℥ij. Gruel.

12 noon. Temperature $105\frac{2}{5}$°. Gruel.

2 P.M., ℥j. rum in gruel.

14th, 6 A.M., temperature $103\frac{3}{5}$°.

7.30 A.M. ℞.—Sodæ salicyl., ʒj.; rum, ℥ij. Gruel. Ate some food; pulse 96, but strong; though much reduced, looks lively; feces yellow; urine clear.

6 P.M., temperature 105°. Administered rum in gruel.

15th, 6 A.M., temperature $102\frac{3}{5}$°; gave gruel.

8 A.M. ℞.—Tr. ferri perchlor., ʒij.; quinine sulph., ʒj.; sprts.

chloroform, ℥j. Gruel. Looks much better, eating mashes and hay and grass.

16th, 6 A.M., temperature 100°.

8 A.M. ℞.—Above repeated. From this on the cow made progress toward recovery.

In perusing the temperatures it is most striking to note the fluctuations. Even for ten days after the 16th I had to give her vegetable and mineral tonics of pulv. anise, fenugreek, gentian, quinine, and sulphate of iron. Before her illness she gave ten quarts of milk a day, now she is giving five and one-half quarts; and after her illness the hair of her tail dropped out.

My other case of red water only lived for thirty hours. I found her at 8 A.M. very weak, with a temperature of $107\frac{2}{5}$°; loose bowels, claret-colored urine, which caused pain to pass; cow rapidly sank and died. This cow was only on the island a week.

What can you assign as the cause of this red water fever? All the cows have been fed and cared alike, and in my cases, with the exception of the first case, which I thought might have been caused by the boy bedding her up with mildewed grass; the other was fed and housed in the same manner as twelve others. I may add that "red water" fever is a most serious disorder among imported cattle. Whatever the cause, the chief organs affected seem to be the liver and kidneys.

Any suggestion you may feel inclined to make will be most acceptable. I was called in to see two cows whose temperatures fluctuated between 103° and 106°, both imported and recently calved. I brought their temperatures down to 102° for a day or two, but it soon went up again. These two cows feed well now, and are giving a constant supply of twelve quarts of milk daily. I suspected tuberculosis, but there was no abnormal sound on auscultation, and no cough. Is this not strange? It is in my experience.

